# TLR2-Dependent Induction of IL-10 and Foxp3^+^CD25^+^CD4^+^ Regulatory T Cells Prevents Effective Anti-Tumor Immunity Induced by Pam2 Lipopeptides *In Vivo*

**DOI:** 10.1371/journal.pone.0018833

**Published:** 2011-04-20

**Authors:** Sayuri Yamazaki, Kohei Okada, Akira Maruyama, Misako Matsumoto, Hideo Yagita, Tsukasa Seya

**Affiliations:** 1 Department of Microbiology and Immunology, Graduate School of Medicine, Hokkaido University, Sapporo, Japan; 2 Department of Immunology, Juntendo University School of Medicine, Tokyo, Japan; Centre de Recherche Public de la Santé (CRP-Santé), Luxembourg

## Abstract

16 S-[2,3-bis(palmitoyl)propyl]cysteine (Pam2) lipopeptides act as toll-like receptor (TLR)2/6 ligands and activate natural killer (NK) cells and dendritic cells (DCs) to produce inflammatory cytokines and cytotoxic NK activity *in vitro*. However, in this study, we found that systemic injection of Pam2 lipopeptides was not effective for the suppression of NK-sensitive B16 melanomas *in vivo*. When we investigated the immune suppressive mechanisms, systemic injection of Pam2 lipopeptides induced IL-10 in a TLR2-dependent manner. The Pam2 lipopeptides increased the frequencies of Foxp3^+^CD4^+^ regulatory T (T reg) cells in a TLR2- and IL-10- dependent manner. The T reg cells from Pam2-lipopeptide injected mice maintained suppressor activity. Pam2 lipopeptides, plus the depletion of T reg with an anti-CD25 monoclonal antibody, improved tumor growth compared with Pam2 lipopeptides alone. In conclusion, our data suggested that systemic treatment of Pam2 lipopeptides promoted IL-10 production and T reg function, which suppressed the effective induction of anti-tumor immunity *in vivo*. It is necessary to develop an adjuvant that does not promote IL-10 and T reg function in vivo for the future establishment of an anti-cancer vaccine.

## Introduction

Foxp3^+^CD25^+^CD4^+^regulatory T (T reg) cells constitute about 5–10% of peripheral CD4^+^T cells and control immunological self-tolerance and tumor immunity [1], [2]. T reg cells directly infiltrate the tumor and suppress effector cells [3]–[5]. T reg cells are also induced from non-T reg cells in the draining lymph nodes of tumor-bearing mice by transforming growth factor (TGF)-β producing dendritic cells (DCs) [6]. Effective anti-tumor immunity is induced by depletion of T reg cells with anti-CD25 monoclonal antibody (mAb) [7]–[9], or blockade of T reg function with anti-CTLA-4 mAb [10]–[12] or anti-GITR mAb [3]. Specific depletion of T reg cells using mice that express diphtheria toxin receptor under the control of the Foxp3 locus induced tumor regression [4], [13]. Therefore, strategies are required to abolish the T reg-induced tolerance that suppresses tumor immunity, thereby establishing an effective anti-tumor immune response.

To overcome the immune suppression mediated by T reg cells in cancer, activation of DCs with adjuvants is required [14], [15]. Adjuvants are mainly targeted to pattern recognition receptors, such as Toll like receptor (TLR) ligands on DCs. To date, cancer vaccine adjuvants have included various TLR agonists such as TLR3, TLR4, TLR5, TLR7 and TLR9 [16], [17]. DCs stimulated by lipopolysaccharide (LPS), a TLR4 agonist, were found to expand functional T reg cells [18], [19]. Hence, it is critical to identify the optimal adjuvants that mature DCs but have less potential to expand T reg cells. However, it is unclear how adjuvants differently affect T reg cell survival and function.

The Bacillus Calmette-Guerin-cell wall skeleton (BCG-CWS) is a TLR2 agonist [20] and has been used as an effective adjuvant for cancer for almost 40 years [16], [21]. However, its clinical usage is limited since BCG-CWS is a large molecular complex unable to be chemically synthesized with full activity. The anti-cancer activity of BCG-CWS operates partly through TLR2 signal [22]–[24], hence, we investigated the adjuvant activity of synthetic TLR2/TLR6 ligands derived from *Staphylococcus aureus*, 16 S-[2,3-bis(palmitoyl)propyl]cysteine (Pam2) lipopeptides. We have previously reported that Pam2 lipopeptides activate DCs and natural killer (NK) cells to produce interferon (IFN)- γ and killer activity *in vitro* [25] and that local injection of Pam2 lipopetides with RGDS peptides, plus tumor extract, could inhibit tumor growth [26].

**Figure 1:**
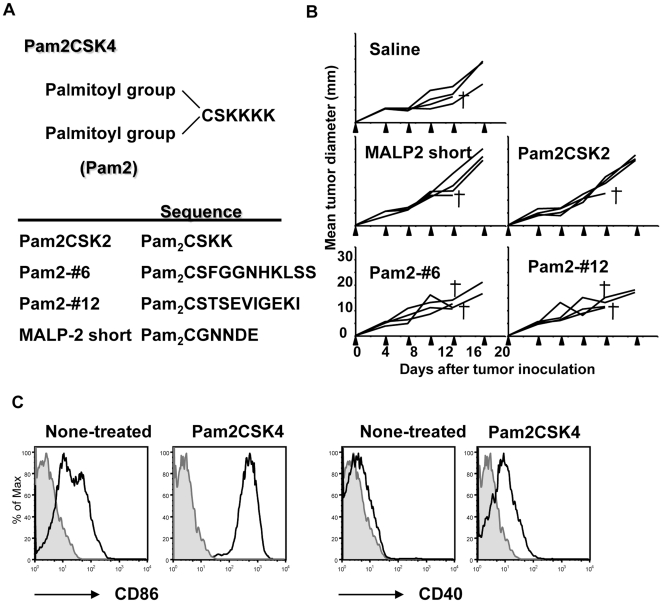
Pam2 lipopeptides do not induce effective anti-tumor immunity. (A) Structures of the Pam2 lipopeptides are shown. (B) Mice were injected with B16D8 melanoma cells (2×10^5^) on back. The mice were injected s.c. into their footpad with the indicated Pam2 lipopeptides (10 nmol) or saline twice a week, as indicated by arrows, starting from day 0. Tumor growth was monitored in a blind manner. A cross indicates the death of one mouse. One of two experiments is shown. (C) Mice were injected with the indicated Pam2 lipopeptides (10 nmol) or saline. After 12–16 hours, spleen DCs were analyzed by flow cytometry. Plots were gated on CD11c^+^ cells. One of two experiments is shown.

Here, we tested if systemic injection of Pam2 lipopeptides in mice could induce an effective anti-tumor immune response. The Pam2 lipopeptides have two palmitoyl-bases attached to different peptide sequences (Fig. 1A) and the peptide portion determines the activity of the TLR2 agonist [25]. We selected the most effective TLR2 activators among the 20 Pam2 lipopeptides [25] and investigated the corresponding anti-tumor response *in vivo*. In contrast with the *in vitro* results, systemic injection of Pam2 lipopeptides did not induce regression of NK-sensitive melanomas. Pam2 lipopeptides induced IL-10 and the expansion of T reg cells *in vivo* in a TLR2-dependent manner. We also found that the depletion of T reg cells by treatment with an anti-CD25 mAb before Pam2 lipopeptide injection, suppressed the tumor growth compared with Pam2-lipopeptide injection alone. These data suggested that systemic injection of Pam2 lipopeptides induced IL-10 and T reg cells, preventing effective tumor immunity *in vivo*. Our findings demonstrate the importance of studying the effects on T reg cells *in vivo* prior to the development of adjuvants.

## Results

### Systemic injection of Pam2 lipopeptides did not induce tumor growth retardation

To examine the anti-tumor effect of the Pam2 lipopeptides *in vivo*, mice were injected subcutaneously (s.c.) with NK-sensitive B16D8 melanomas into their back [22] and were treated with Pam2 lipopeptides twice a week (Fig. 1B). We selected four kinds of Pam2 lipopeptides, as shown in Fig. 1A, because they strongly activated NK cells through DCs and induced cytotoxic activity *in vitro* [25]. To our surprise, although the Pam2 lipopeptides activated NK cells *in vitro* [25], we did not observe effective anti-tumor response *in vivo* (Fig. 1B). To exclude the possibility that Pam2 lipopeptides were not distributed systemically, we investigated the activation of spleen DCs and NK cells by flow cytometry. The injection of Pam2 lipopeptides up-regulated CD86 and CD40 on splenic DCs (Fig. 1C). Similarly, CD69 was up-regulated in splenic NK cells (Supplemental Fig. S1). Thus, systemic injection of Pam2 lipopeptides was able to activate DCs and NK cells in the spleen, but did not induce effective anti-tumor responses *in vivo*.

### Pam2 lipopeptides induce IL-10 *in vitro* and *in vivo* in a TLR2-dependent manner

To investigate why Pam2 lipopeptides could not induce effective anti-tumor responses against NK-sensitive tumors *in vivo*, we investigated whether Pam2 lipopeptides could activate suppressive factors, such as IL-10 and T reg -related molecules. For this experiment, we mainly used a representative Pam2 lipopeptide, Pam2CSK4, since Pam2CSK4 could activate DCs as well as other tested Pam2 lipopeptides *in vitro* [25].

**Figure 2:**
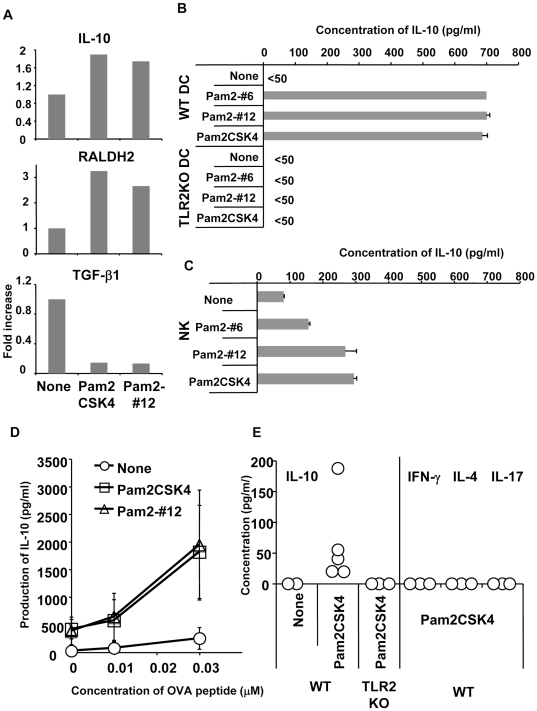
Pam2 lipopeptides induce IL-10 and retinal dehydrogenase. (A) Spleen DCs from B6 mice were cultured with or without 100 nM of Pam2CSK4 or Pam2-#12. After four hours, total RNA was prepared and real-time PCR was performed. Expression of each sample was normalized to GAPDH mRNA expression and fold increases of each sample were calculated to the expression levels at 0 hours. One of two experiments is shown. (B) BM-DCs (1×10^5^) from wild type (WT) or TLR2KO mice were cultured with or without 100 nM of Pam2-#6, Pam2-#12 and Pam2CSK4 for 24 hours. The culture supernatants were measured for IL-10. One of two experiments is shown. (C) NK cells (2×10^5^) from spleens were cultured with 100 nM of Pam2-#6, Pam2-#12 and Pam2CSK4 for 24 hours. The culture supernatants were measured for IL-10. One of two experiments is shown. (D) OT II CD4^+^ T cells (5×10^4^) were cultured with spleen DCs (5×10^4^) with or without 100 nM of Pam2CSK4 or Pam2-#12 and the various doses of OVA peptide. After five days, supernatants were measured for IL-10. The means +/- SDs from two separate experiments is shown. (E) WT or TLR2KO mice were i.p. injected with 10 nmol Pam2CSK4 and next day serum was measured for the indicated cytokine concentrations.

When the mRNA levels from DCs stimulated with or without Pam2 lipopeptides were analyzed, Pam2 lipopeptides up-regulated retinal dehydrogenase 2 (RALDH2) and IL-10. RALDH2 in DCs activates retinoic acid, which is an important cofactor for TGF-β1 to induce Foxp3 [27], [28]. However, Pam2 lipopeptides did not up-regulate the mRNA of TGF-β1 (Fig. 2A).

To confirm whether IL-10 protein is produced from DCs, we stimulated DCs with Pam2 lipopeptides *in vitro* for 24 hours and the concentration of IL-10 in the supernatants was measured by the ELISA. Bone-marrow derived DCs **(**BM-DCs) stimulated by Pam2 lipopeptides produced IL-10 (Fig. 2B). IL-10 was also produced by Pam2 lipopeptide-stimulated DCs from the spleen (data not shown). When DCs from TLR2- knockout (TLR2KO) mice were cultured with Pam2 lipopeptides, the production of IL-10 was not detected (Fig. 2B). Hence, IL-10 production was TLR2 dependent. Interestingly, we also found that Pam2 lipopeptides induced IL-10 production from NK cells (Fig. 2C).

To determine whether CD4^+^ T cells produced IL-10 in the presence of Pam2 lipopeptides, OT II ovalbumin (OVA) transgenic CD4^+^ T cells were cultured with DCs along with various doses of OVA peptide, with or without Pam2 lipopeptides (Fig. 2D). In the presence of Pam2 lipopeptides, more IL-10 was produced in the culture supernatants when OT II CD4^+^ T cells were cultured with DCs and antigen (Fig. 2D). Importantly, IL-10 production was increased in an antigen-dose dependent manner (Fig. 2D).

Next, we analyzed the concentration of IL-10 in the serum of Pam2 lipopeptide-treated mice (Fig. 2E). When serum was taken at one day after Pam2CSK4 injection, significant amounts of IL-10 were detected (Fig. 2E), however, Th1, Th2 and Th17 cytokines were not detected (Fig. 2E). IL-10 production in serum was confirmed to be TLR2 dependent because we could not detect IL-10 in Pam2CSK4-treated TLR2KO mice (Fig. 2E). Taken together, these results indicated that Pam2 lipopeptides induce IL-10 both *in vitro* and *in vivo* in a TLR2-dependent manner, which might play a role in suppressing tumor immunity induced by Pam2 lipopeptides.

### Systemic injection of Pam2 lipopeptides expands T reg cells through the TLR2 dependent production of IL-10

**Figure 3:**
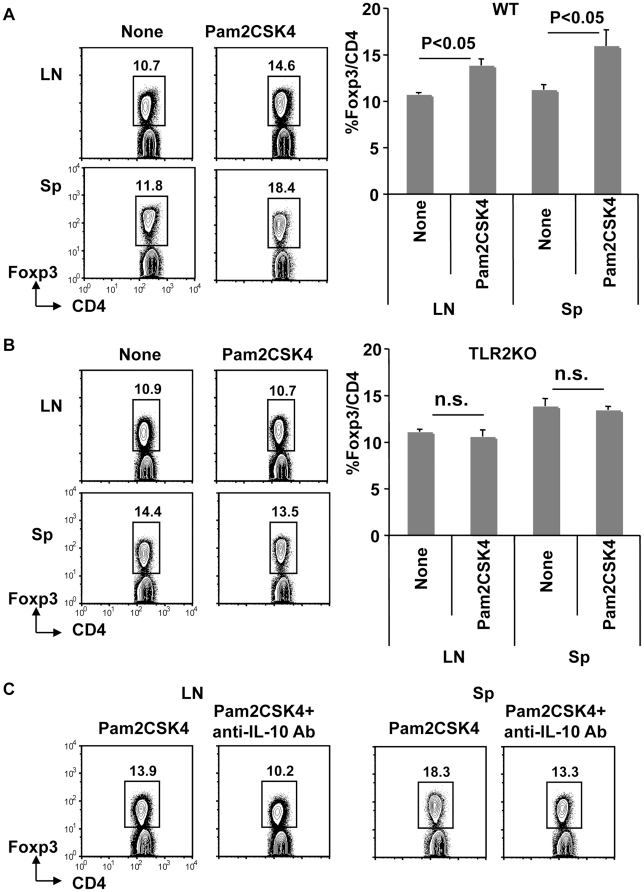
Systemic injection of Pam2CSK4 expands Foxp3^+^ T reg cells in a TLR2- and IL-10-dependent manner. (A) WT mice were i.p. injected with Pam2CSK4 (10 nmol). After three days, spleen (Sp) and lymph node (LN) cells were analyzed for the expression of Foxp3. The plots were gated on CD4^+^ T cells. One of four experiments is shown for the FACS plots. The image summarizes the results of four separate experiments. P value is derived from the student's-t test. (B) As in (A), but TLR2KO mice were injected with Pam2CSK4. One of two experiments is shown for the FACS plots. The image summarizes the results from two separate experiments. N.s. stands for not significant according to the student's-t test. (C) As in (A), but mice were i.p. injected with Pam2CSK4 with or without 200 µg of anti-IL-10 mAb. One of two experiments is shown.

Since Pam2 lipopeptides induce IL-10, we investigated whether systemic injection of Pam2 lipopeptides could affect T reg cell frequencies. IL-10 produced by zymosan plays a role in inducing T reg cells [29]. We found that the frequency of Foxp3^+^ T reg was increased in the spleen and lymph nodes at day 3 after systemic injection of Pam2CSK4 (Fig. 3A). The frequency of T reg cells had returned to normal by day 7 after Pam2CSK4 injection (Supplemental Fig. S2). The increase of T reg cells was dependent on TLR2 because T reg cells were not increased in TLR2KO mice injected with Pam2CSK4 (Fig. 3B).

To investigate whether the increase of Foxp3^+^ T reg is dependent on the IL-10 produced by Pam2CSK4, mice were injected with neutralizing anti-IL-10 mAb (JES5-2A5) and Pam2CSK4 (Fig. 3C). Control mice injected with anti-IL-10 mAb alone or untreated mice were not included in this experiment, however, the frequency of Foxp3^+^ T reg cells in the mice injected with anti-IL-10 Ab alone would be expected be similar to that of naïve mice since it is reported that the frequency of Foxp3^+^ T reg cells is not affected in the spleen of IL-10 [30] or IL-10 receptor β knockout mice [31]. After three days, co-administration of anti-IL-10 mAb blocked the increase of T reg cells after Pam2CSK4 injection (Fig. 3C).

Therefore, Pam2 lipopeptides expand Foxp3^+^ T reg cells at day 3 after systemic injection in a TLR2- and IL-10 dependent manner.

### T reg cells from Pam2 lipopeptide-treated mice have suppressive activity

**Figure 4:**
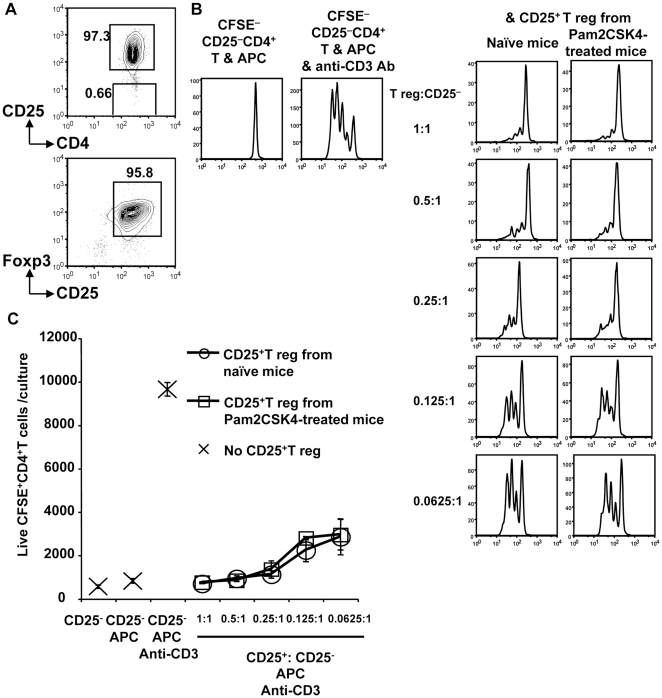
T reg cells from Pam2CSK4-treated mice maintain suppressive activity. (A)CD25^+^CD4^+^ T cells purified by flow cytometry were further fixed and stained with Foxp3. FACS plots were gated on CD4^+^ T cells. One of three similar experiments is shown.(B) B6 mice were i.p. injected with Pam2CSK4 (10 nmol) on days 0, 3 and 7. On day 14, CD25^+^CD4^+^ T cells purified as in (A) were used for the suppression assay. CFSE-labeled CD25^-^ CD4^+^ T cells (5×10^4^) were stimulated with irradiated spleen antigen presenting cells (1×10^5^) with or without 5% anti-CD3 mAb supernatant. The purified CD25^+^CD4^+^ T cells from naïve mice or Pam2CSK4-treated mice were added at the indicated ratio. After three days, cells were stained with CD4 and analyzed with CFSE dilution. Dead cells were eliminated by TOPRO-3. One of three similar experiments is shown. (C) As in (B), but the numbers of live CFSE^+^ CD4^+^T cells per culture were plotted. One of three similar experiments is shown.

Next, we investigated the suppressive function of T reg cells in Pam2 lipopeptide-treated mice. We purified CD25^+^CD4^+^ T cells from naive mice or Pam2 lipopeptide-treated mice by flow cytometry (Fig. 4A). The frequency of Foxp3^+^CD4^+^ T cells in the purified CD25^+^CD4^+^ T cells from naïve mice or Pam2 lipopeptide-injected mice was always >95%, as shown in Fig. 4A. The purified CD25^+^CD4^+^ T cells were used for the classical *in vitro* suppression assay [32]. We found that the CD25^+^ T reg cells from Pam2CSK4-treated mice suppressed the proliferation of CD25^-^ CD4^+^ T cells from naïve mice to a similar degree compared with the CD25^+^ T reg from naïve mice (Fig. 4B, C). This indicated that Pam2 lipopeptides maintain T reg cell function *in vivo*.

### Depletion of T reg cells improves the anti-tumor response by systemic injection of Pam2 lipopeptides

**Figure 5:**
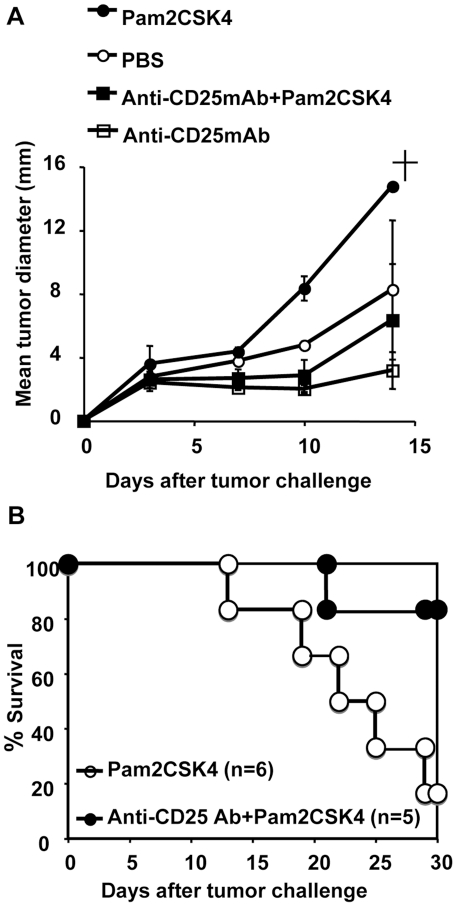
NK-sensitive tumors grow slowly when Pam2CSK4 is injected into T reg-depleted mice. (A) B6 mice were i.p. injected with 500 µg of anti-CD25mAb (PC61) on day -3. The mice were injected with B16D8 melanoma cells (2×10^5^) into their back on day 0. Pam2CSK4 (10 nmol) or PBS was injected twice a week from day 0 to day 14. Tumor growth was monitored twice a week. n = 3 for each group. A cross indicates the death of one mouse. One representative experiments from two similar experiments is shown. (B) As in (A), but the survival curve summarized from two separate experiments is shown.

To determine whether systemic injection of Pam2 lipopeptides activates the function of T reg cells and suppresses anti-tumor responses against NK-sensitive tumors *in vivo*, we used an anti-CD25mAb (PC61) to deplete T reg cells *in vivo* before challenge with Pam2 lipopeptide and tumor cells [7]–[9]. Mice were injected with anti-CD25 mAb on day −3 and challenged with B16D8 melanoma cells on day 0, with or without Pam2CSK4 (Fig. 5A). As previously reported, depletion of T reg cells alone induced growth retardation of tumors [7]–[9]. Tumor growth was slightly promoted by Pam2CSK4 injection alone (Fig. 5A). However, the tumor growth in mice treated with anti-CD25 mAb plus Pam2CSK4 was slower than in mice treated with Pam2CSK4 alone (Fig. 5A, B). These results suggested that the presence of T reg cells suppressed effective anti-tumor responses after systemic injection of Pam2 lipopeptides.

## Discussion

Here, we showed that systemic injection of Pam2 lipopeptides did not induce effective tumor immunity presumably because of the induction of IL-10 and T reg cells. To treat cancer, it is necessary to develop new adjuvants to activate immunity in immune-suppressed patients. Adjuvant activity is generally screened by analyzing its effect on effector cells such as NK cells and CD8^+^ cytotocxic T cells. However, our results indicated that it is also important to investigate the activity of adjuvants on suppressive factors, such as IL-10 and T reg cells, particularly *in vivo*.

IL-10 is a key cytokine for IL-10 producing Tr1 regulatory T cells [33], and has also been shown to be an important cytokine for Foxp3^+^ T reg cells. IL-10 production by Foxp3^+^ T reg cells is required for the prevention of colitis [34], [35]. The specific deletion of IL-10 in Foxp3^+^ T reg cells in mice induces inflammation especially in the intestine, indicating that IL-10 derived from T reg cells plays a critical role in controlling colitis [35]. Furthermore, M. Kronenberg and his colleagues recently found that IL-10 secreted by other cells is needed for T reg cells to sustain expression of Foxp3 and prevent colitis [31]. This indicated that IL-10-enriched environments are preferable for Foxp3^+^ T reg cells to exert their suppressive function *in vivo*. Here we have shown that systemic injection of Pam2 lipopeptides induces IL-10-rich environments *in vivo*, which could play a role in promoting T reg cell function.

Our results showed that TLR2-dependent production of IL-10 plays a role in expanding T reg cells *in vivo* (Fig. 3). This is consistent with a recent report by B. Pulendran and his colleagues who showed that TLR2 signaling by zymosan induces IL-10 and retinal dehydrogenase in DCs, which are critical for inducing T reg cells [29]. Zymosan binds to TLR2 and dectin-1 [29]. Our data showed that the TLR2 signal induced by Pam2 lipopeptides has a similar effect to the signal induced by zymosan. The TLR2 signal induced by zymosan results in the active suppression of experimental autoimmune encephalomyelitis (EAE) [29]. Furthermore, various TLR signals prevent the development of autoimmune type 1 diabetes in non-obese diabetic mice [36]. Our results showed that systemic injection of Pam2 lipopeptides was ineffective at inducing tumor immunity. However, it is possible that the Pam2 lipopeptides might be useful to inducing tolerance in the case of autoimmunity, allergy or transplant rejection.

In addition to the evidence that IL-10 produced in response to the TLR2 signal affects Foxp3^+^ T reg cell function, the TLR2 signal can also directly act on T reg cells and promotes their survival [37]. Taken all together, although TLR2 activation by Pam2 lipopeptides is able to induce inflammatory cytokines and activate NK cells *in vitro* [25], the systemic injection of Pam2 lipopeptides as cancer adjuvants is ineffective at abolishing immune suppression. Whereas, the effective cancer adjuvant, BCG-CWS, activates not only TLR2, but also TLR4 and NOD2 receptors [23], [38]. TLR2 activation by Pam2 lipopeptides could activate T reg cells *in vivo* and the T reg cells could suppress NK function and activation [39]–[41]. Our preliminary experiments showed that T reg cells actually suppress IFN-γ production from NK cells stimulated with DCs plus Pam2CSK4 (S.Y., K.O., T.S., unpublished data). Here we showed that depletion of T reg cells with adjuvant might be one potential strategy to cancel the effect of activating suppressive factors by Pam2 lipopeptides.

We also found that Pam2 lipopeptides induce IL-10 production from NK cells *in vitro* (Fig. 2C). It has been known for over a decade that NK cells produce IL-10 [42]–[44]. Recent reports showed that IL-10 produced by NK cells play an important role in controlling T cell responses [45], [46] and anti-inflammatory responses [47]. Moreover, IL-10 enhances the killing by NK cells of autologous antigen presenting cells [48], [49]. These reports suggested that IL-10-stimulated NK cells could kill autologous macrophages and DCs, which may result in suppressing effective anti-tumor immunity. Therefore, it is possible that systemic injection of Pam2 lipopeptides in our system may induce IL-10 from NK cells and suppress anti-tumor response *in vivo*.

In contrast to the systemic injection of Pam2 lipopeptides, local injection of Pam2 lipopeptides was effective at suppressing tumor growth when the Pam2 lipopeptide was fused to RGDS-integrin peptides and injected around the tumor with tumor extracts [26]. This was probably effective for a few reasons:1) the peptide part of the Pam2 lipopeptide was fused with RGDS, which could promote the binding of Pam2 lipopeptides to DCs; 2) local injection of Pam2 lipopeptides around the tumor may be different from systemic injection of Pam2 lipopeptides in terms of inducing IL-10 and T reg cells. Other literature has also indicated that local administration of Pam2 lipopeptides could be effective for cancer [50], [51]. The differential effect on inducing IL-10 and T reg cells between local administration and systemic injection of adjuvants should be investigated further in future studies.

The literature on TLR2 signaling and T reg cells is controversial. Some groups reported that T reg cells temporally lost their suppressive capacity in the presence of the TLR2/TLR1 ligand Pam3CSK4, which contains 3-palmitoyl bases [52], [53]. However, a recent report from E. Shevach and his colleagues showed that the presence of Pam3CSK4 in the culture actually maintained the suppressive function of T reg cells and promoted their survival [37]. This discrepancy might be caused by the use of CD25^+^ CD4^+^ T cells contaminated with Foxp3^-^CD25^+^CD4^+^ T cells, and Foxp3-GFP reporter mice [37]. Contamination of Foxp3^-^CD25^+^CD4^+^ T cells could affect the results, especially when TLR2 ligands were continuously present in the culture, since TLR2 is also expressed on activated effector T cells. In this report, we stimulated T reg cells with Pam2 lipopeptides *in vivo* and purified T reg cells as CD25^+^ CD4^+^ T cells because Foxp3-GFP reporter mice were not available. However, CD25^+^ CD4^+^ T reg cells purified from Pam2CSK4 treated mice were as suppressive as T reg cells from naïve mice (Fig. 4B). This indicated that T reg cells stimulated by TLR2 did not reverse the suppressive function *in vivo*.

To fight to cancer, it is very important to develop an adjuvant to activate immunity. However, it is also crucial to consider the effect of adjuvants on suppressive factors such as IL-10 and T reg cells. The combination of adjuvant and blockade of IL-10 or T reg cell function might prove a successful strategy for improving cancer vaccines.

## Materials and Methods

### Mice

C57BL6J (B6) mice and CB17SCID mice were obtained from Japan Clea (Tokyo, Japan). TLR2KO mice were provided by Dr. Shizuo Akira (Osaka University, Osaka, Japan). OT II OVA CD4 transgenic mice were kindly provided from Dr. Kazuya Iwabuchi (Kitasato University, Kanagawa, Japan). The mice were maintained in the Hokkaido University Animal Facility (Sapporo, Japan) in specific pathogen free condition. All experiments used mice that were between 6–12 weeks-of-age at the time of first procedure. All mice were used according to the guidelines of the institutional animal care and use committee of the Hokkaido University, who approved this study as ID number: 08-0243, “ Analysis of immune modulation by toll-like receptors”.

### Antibodies and reagents

PE-conjugated CD25 (PC61), Alexa-488 conjugated anti-CD25 (7D4), FITC or APC conjugated CD4 (RM4-5), CD11c, NK1.1, purified anti-CD16/CD32 (2.4G2), purified anti-CD3 (2C11), and isotype antibodies were obtained from Biolegend (San Diego, CA, USA). Anti-CD11c, anti-NK and streptavidin microbeads were purchased from Miltenyi Biotec (Gladbach, Germany). Carboxyfluorescein diacetate succinimidyl ester (CFSE) and TOPRO-3 were from Molecular Probes (Eugene, OR, USA). The anti-mouse Foxp3 (FJK-16s) staining kit was from eBioscience (San Diego, CA, USA). Purified anti-CD25 (PC61) mAb was a gift from Dr. Ralph Steinman (The Rockefeller University, NY, USA) and anti-CD25 hybridoma cells were from Dr. Jun Shimizu (Kyoto University, Kyoto, Japan). Some of the anti-CD25 (PC61) mAb was produced in CB17SCID mice in our animal facility and purified by ammonium sulfate precipitation. Purified anti-IL-10 mAb (JES5-2A5) was prepared as described previously [54]. Pam2CSK4, Pam2CSK2 and MALP2 short lipopeptides were synthesized by Biologica Co. Ltd (Nagoya, Japan). Pam2-#6 and Pam2-#12 were from Dr. Yukari Fujimoto and Dr. Koichi Fukase (Osaka University, Osaka, Japan).

### Cell isolations

CD4^+^ T cells were first negatively separated by MACS beads from lymph nodes and spleen cell suspensions (>90%) (Miltenyi Biotech) and T reg cells were further purified by a FACS Aria II (BD Bioscience, Franklin Lakes, NJ, USA). Spleen CD11c^+^ DCs were selected with anti-CD11c beads (Miltenyi Biotech). Bone marrow DCs **(**BM-DCs) were cultured with GM-CSF as previously described [22]. NK cells were purified from spleen by anti-NK beads (Miltenyi Biotech). To analyze the activation of DCs and NK cells *in vivo* by Pam2 lipopeptides, 10 nmol of Pam2 lipopeptides was subcutaneously (s.c.) or intraperitoneally (i.p.) and 12–16 hours later, the spleen was analyzed by flow cytometry. Both routes of injection gave similar results.

### Measuring cytokine production

DCs or NK cells were stimulated with 100 nM of Pam2 lipopeptides for 24 hours and the supernatants were measured for IL-10 by ELISA (eBiosciences). CD4^+^ T cells from OT II transgenic mice were cultured with spleen DCs with or without 100 nM of Pam lipopeptides at the various doses of OVA peptide for five days. The supernatants were measured for IL-10 by ELISA. Serum from Pam2 lipopeptides treated mice or control mice were taken one day after i.p. injection and were measured for IL-10, INF-γ, IL-4 and IL-17 by Cytometric Bead Array (BD Bioscience). Analysis with the Cytometric Bead Array was performed according to the manufacturer's instructions.

### Quantitative PCR

Total RNA was isolated with TRIzol (Invitrogen by life technologies, Carlsbad, CA, USA), and reversed transcribed by High Capacity cDNA Transcription Kit (ABI by life technologies, Carlsbad, CA, USA) according to manufacturer instructions. The qPCR was performed with the Step One Real-Time PCR system (ABI). The primers used for real-time PCR have been reported previously [29].

### *In vivo* tumor challenge

Mice were s.c. injected with 2−3×10^5^ B16D8 cells into the back. B16D8 melanoma is a NK-sensitive B16 melanoma cell line, which we have previously established [22]. The tumor growth was monitored twice a week. Sometimes mice were pre-treated with 500 µg of anti-CD25 mAb three days before tumor challenge. Then, 10 nmol of Pam2 lipopeptides or control saline was s.c injected into footpad or i.p. injected twice a week. The both routes of injection gave similar results.

### *In vitro* suppression assay using T reg cells

The classical *in vitro* suppression assay was performed as previously described [32], [55]. Briefly, CD25^+^CD4^+^ T cells were purified by flow cytometry and used as suppressor cells. CFSE-labeled CD25^-^ CD4^+^ T cells or CD4^+^ T cells were stimulated with or without anti-CD3 mAb (2C11) and 15–20 Gy irradiated spleen cells. Various numbers of suppressor cells were added to the culture. After three day culture, cells were stained with CD4 –PE and dead cells were gated out with TOPRO-3 (Molecular Probes). All cells in each culture were acquired using the FACS calibur (BD Bioscience) to have the cell yield and number of live CFSE^+^ cells/culture was calculated. Analysis was performed with Flowjo software (TreeStar, USA).

## Supporting Information

Figure S1 **NK cells up-regulates CD69 after systemic injection of Pam2 lipopeptides.** Mice were subcutaneously injected with the indicated Pam2 lipopeptides (10 nmol) or saline. After 16 hours, splenic NK cells were analyzed by flow cytometry. Plots were gated on NK1.1^+^ cells.
(TIF)

Figure S2 **The frequency of T reg cells returns to normal at day 7 after systemic injection of Pam2 lipopeptides.** WT mice were i.p. injected with Pam2CSK4 (10 nmol). After seven days, spleen (Sp) and lymph node (LN) cells were analyzed for the expression of Foxp3. The plots were gated on CD4^+^ T cells. One of two experiments is shown for the FACS plots.
(TIF)
