# Supplementary figures and images for: TLR2-Dependent Induction of IL-10 and Foxp3+CD25+CD4+ Regulatory T Cells Prevents Effective Anti-Tumor Immunity Induced by Pam2 Lipopeptides In Vivo
Source: PLoS One. 2011 Apr 20;6(4):e18833. doi: 10.1371/journal.pone.0018833 (PMC3080372; doi:10.1371/journal.pone.0018833)

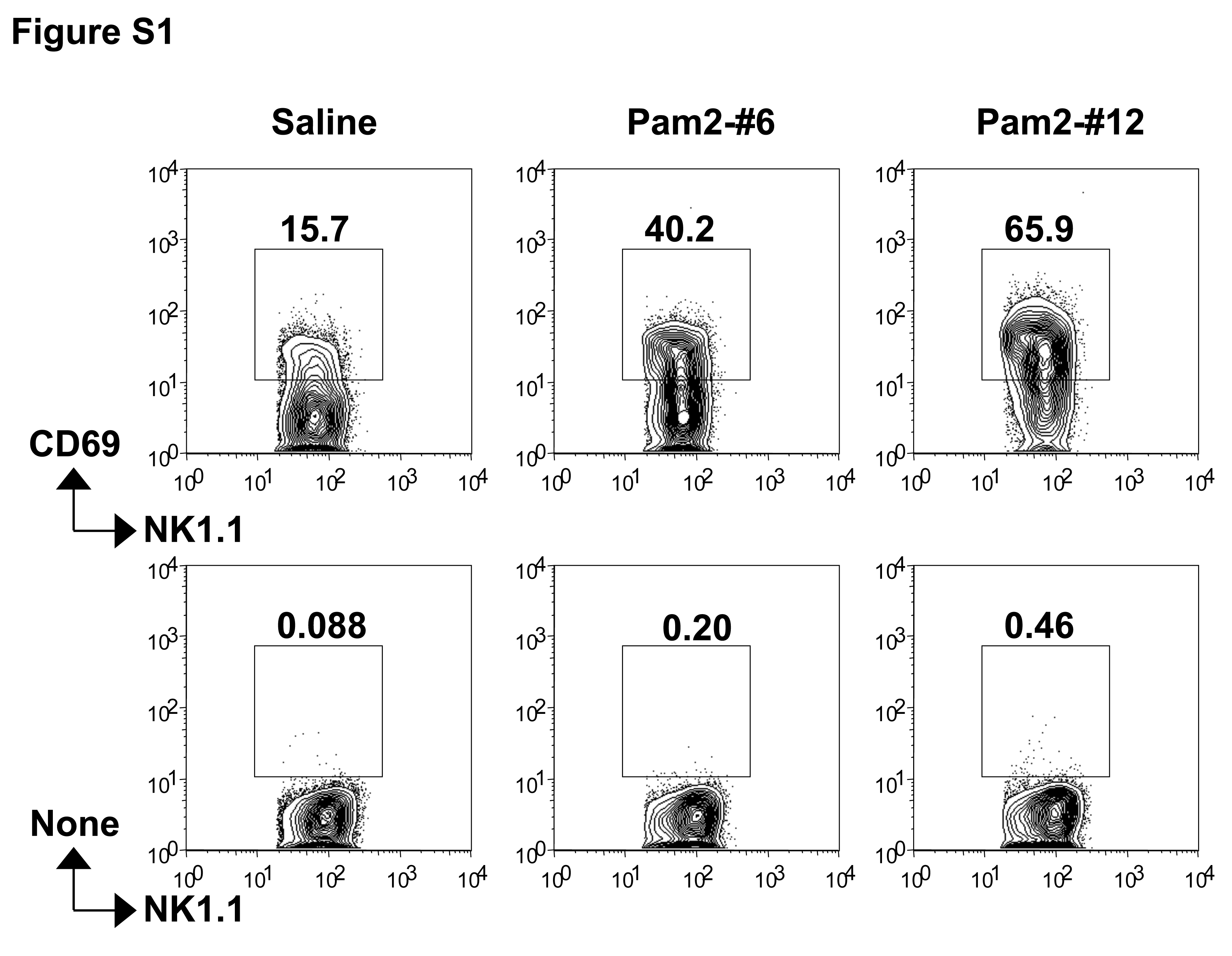

Supplement: Figure S1 — NK cells up-regulates CD69 after systemic injection of Pam2 lipopeptides. Mice were subcutaneously injected with the indicated Pam2 lipopeptides (10 nmol) or saline. After 16 hours, splenic NK cells were analyzed by flow cytometry. Plots were gated on NK1.1+ cells. (TIF) [file pone.0018833.s001.tif]

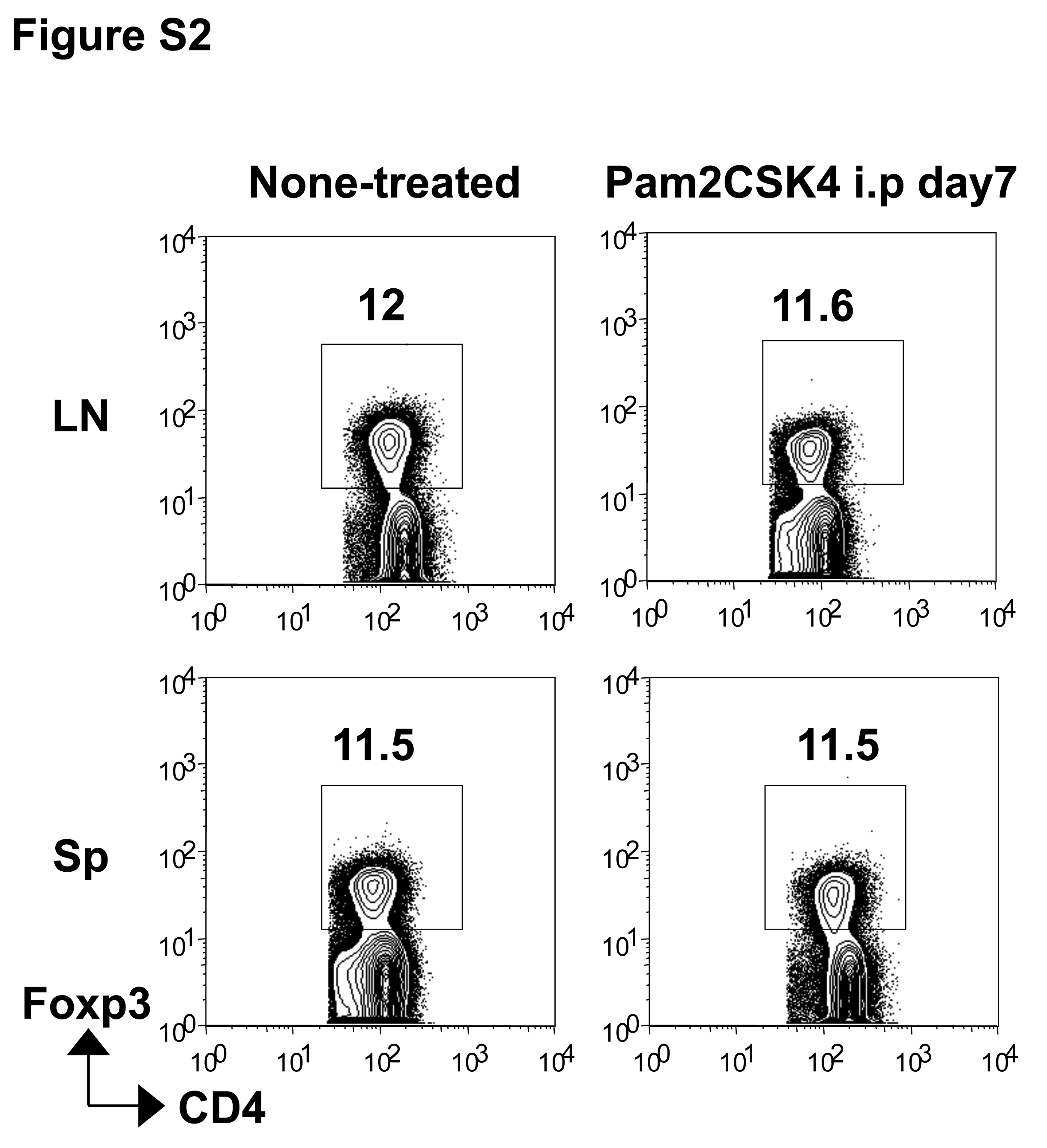

Supplement: Figure S2 — The frequency of T reg cells returns to normal at day 7 after systemic injection of Pam2 lipopeptides. WT mice were i.p. injected with Pam2CSK4 (10 nmol). After seven days, spleen (Sp) and lymph node (LN) cells were analyzed for the expression of Foxp3. The plots were gated on CD4+ T cells. One of two experiments is shown for the FACS plots. (TIF) [file pone.0018833.s002.tif]
